# Polarization image segmentation of radiofrequency ablated porcine myocardial tissue

**DOI:** 10.1371/journal.pone.0175173

**Published:** 2017-04-05

**Authors:** Iftikhar Ahmad, Adam Gribble, Iqbal Murtza, Masroor Ikram, Mihaela Pop, Alex Vitkin

**Affiliations:** 1 Department of Physics and Applied Mathematics, Pakistan Institute of Engineering and Applied Science (PIEAS), Nilore, Islamabad, Pakistan; 2 Department of Medical Biophysics, University of Toronto, Toronto, Ontario, Canada; 3 Division of Biophysics and Bioimaging, Princess Margaret Cancer Centre, University Health Network, 610 University Avenue, Toronto, Ontario, Canada; 4 Department of Computer and Information Sciences, Pakistan Institute of Engineering and Applied Science (PIEAS), Nilore, Islamabad, Pakistan; 5 Sunnybrook Research Institute, Department of Medical Biophysics, University of Toronto, 2075 Bayview Avenue, Toronto, Ontario, Canada; 6 Department of Radiation Oncology, University of Toronto, 610 University Avenue, Toronto, Ontario Canada; University of Minnesota, UNITED STATES

## Abstract

Optical polarimetry has previously imaged the spatial extent of a typical radiofrequency ablated (RFA) lesion in myocardial tissue, exhibiting significantly lower total depolarization at the necrotic core compared to healthy tissue, and intermediate values at the RFA rim region. Here, total depolarization in ablated myocardium was used to segment the total depolarization image into three (core, rim and healthy) zones. A local fuzzy thresholding algorithm was used for this multi-region segmentation, and then compared with a ground truth segmentation obtained from manual demarcation of RFA core and rim regions on the histopathology image. Quantitative comparison of the algorithm segmentation results was performed with evaluation metrics such as dice similarity coefficient (DSC = 0.78 ± 0.02 and 0.80 ± 0.02), sensitivity (*S*_*n*_ = 0.83 ± 0.10 and 0.91 ± 0.08), specificity (*S*_*p*_ = 0.76 ± 0.17 and 0.72 ± 0.17) and accuracy (*Acc* = 0.81 ± 0.09 and 0.71 ± 0.10) for RFA core and rim regions, respectively. This automatic segmentation of parametric depolarization images suggests a novel application of optical polarimetry, namely its use in objective RFA image quantification.

## Introduction

Radiofrequency ablation (RFA) is an effective treatment for focal arrhythmia, where properly titrated radiofrequency (RF) energy enables controlled destruction of arrhythmogenic regions in myocardium via resistive tissue heating, creating a permanent lesion [1,2]. Spatial overlap of the resultant RFA thermal lesion with the planned treatment geometry is a primary determinant for successful elimination of arrhythmogenic foci [3].

Typically, ablated tissue consists of a lesion core of coagulative necrosis surrounded by a rim region comprised of intermixed viable and non-viable cells [1,4]. The RFA rim region is sometimes hard to differentiate. However, this is essential to identify, since residual viable cells might complicate or resist the desired “electric isolation” in the targeted area, resulting in RFA treatment failure. Further, conduction velocity and electrophysiological properties of the rim region are also altered relative to both healthy tissue and RFA core [5]. Commonly used medical imaging modalities (ultrasound, computed tomography (CT), magnetic resonance (MR)) for RFA lesion assessment can distinguish the lesion core from healthy myocardium, with a limitation that the important ‘rim region’ is relatively unexplored [6,7]. There is thus a need for quantitative imaging methods that can differentiate the rim region consisting of intermixed viable and necrotic cardiomyocytes.

Recently, various optical imaging techniques have been investigated for characterization and subsequent segmentation of cardiac RFA lesions. For example, quantitative evaluation of RFA lesion area by photoacoustic imaging resulted in about 69% area agreement with gross pathology [8]. In addition, optical coherence tomography (OCT) has been explored for qualitative assessment of the RFA lesion spatial extent in swine myocardium; it was observed that untreated tissue exhibited consistent birefringence due to organized myocardium, which was not present in ablated tissue [9,10]. However, these optical imaging techniques (like CT, MR and ultrasound above) focus on delineating the RFA core from the surrounding healthy tissue, while largely ignoring the important, intermediary rim region.

There are different reasons why these techniques have not been used to investigate the rim regions. A major limitation of CT is its limited soft tissue contrast that makes it challenging to quantitatively delineate the relatively small but clinically important rim region. OCT, ultrasound and photoacoustic imaging may be better alternatives. However, to the best of our knowledge past work using these modalities has only focused on binary segmentation of RFA lesion from healthy tissue, and has not investigated the rim region [6,8,9]. MR has been used to visualize the rim of the RFA lesion [11]. However, its high cost, overall complexity, and limited accessibility make it desirable to investigate simpler and more affordable techniques, such as optical polarimetry.

Optical polarimetry has been investigated for assessment of many pathological conditions of biological tissues, such as infarcted and stem cell regenerated myocardium [12,13], structural disorders of the bladder wall in partial bladder outlet obstruction [14–16], and various cancers [17–21]. Recently, polarized light measurements have revealed significant differences in depolarization between RFA core, rim and healthy regions of RF ablated tissue [22]. The current study extends this, focusing on automatic, objective segmentation into these three clinically relevant zones, with applications for focal RF arrhythmia treatments [22].

## Materials and methods

### Sample preparation

RFA lesions were generated *ex vivo* on the left ventricle (LV) endocardium of porcine heart samples (n = 5) using a Navistar catheter (Biosense Webster) coupled to a 460 kHz RF generator (Radiotherapeutics, Biosense, Webster), by applying a power of 20–30 W for 40–60 sec at the tip of the catheter. All hearts were harvested from approximately 2-month old slaughter- house animals weighing around 40 kg. After visual confirmation of lesion formation via gross pathology, the hearts were fixed in 10% formalin for at least 24 hours and 4 mm-thick transverse slices were cut for optical polarimetry measurements (described below). After polarimetric imaging, tissue samples were embedded in paraffin, sliced to 4-μm-thick with a microtome, stained with Masson trichrome, and examined histologically. Digital microscopy images of the stained tissue slides were acquired for subsequent image analysis, pathology delineation, and comparison with polarimetry.

### Polarized light imaging and data analysis

**Fig 1:**
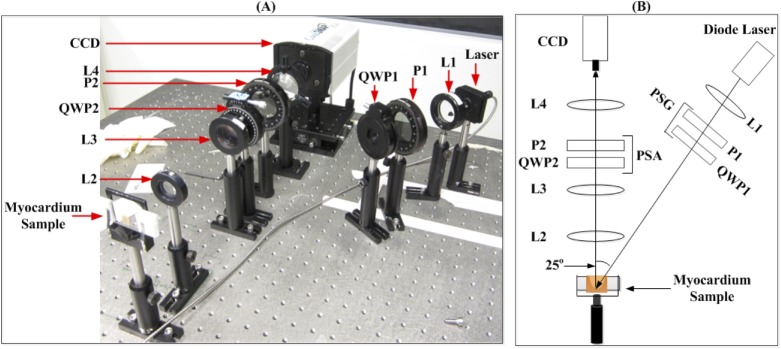
Experimental Setup. (A) Photo of experimental Mueller matrix imaging setup and (B) schematic of (A). Input polarization states are generated using the polarization state generator (PSG), and output polarizations following sample interaction are analyzed with the polarization state analyzer (PSA). P1, P2 = polarizers; QWP1, QWP2 = quarter wave plates; L1, L2, L3, L4 = lenses.

Polarized light imaging was performed in a backscattering geometry (25° off exact backscatter, see Fig 1). This is a more clinically relevant geometry than transmission, which requires illumination and detector to be on opposite sides of the sample, and is limited to thin sample thicknesses (< 2–3 mm). The illumination source was a 635nm diode laser (Thorlabs). Samples were sequentially illuminated by four different polarization states (linear horizontal, vertical, and +45°, and right circular) using a rotatable polarizer P1 and removable quarter wave plate QWP1. For each input polarization state, scattered light following sample interaction was analyzed with six different output polarizations (linear horizontal, vertical, +45°, -45°; right and left circular) using a removable quarter wave plate QWP2, followed by rotatable polarizer P2. A CCD camera recorded each of these 24 (four input x six output) polarization images (CoolSnap K4, Photometrics). The polarization images were then used to calculate tissue Mueller matrices **M** of each pixel in the image, as shown in detail elsewhere [12].

All tissue polarimetric effects are contained in the 16 elements of its Mueller matrix, **M,** in an intermixed form. **M** then has to be further analyzed to isolate the polarization properties of interest (for this study, total depolarization Δ_T_). One way to extract Δ_T_ (along with other properties such as linear retardance, to be investigated in a future study) from the measured **M** is with a technique called polar decomposition [23], which was used here.

### Local fuzzy thresholding algorithm

For easy clinical interpretation of depolarization images, segmentation based on underlying pathology is required. In this study, we propose to use a computer-aided analysis approach. While many computer-aided medical image segmentation techniques are available [24–26], most are rather computationally extensive and application-specific [27,28]. A common and simple segmentation technique, global rigid thresholding, uses cut-off gray level values to classify each pixel into a separate segment. However, incorrect classifications may arise due to gradual transitions between different segments (such as depolarization from RFA core to rim, and from rim to healthy region), image noise and uneven illumination [29]. Part of these problems arises from ignoring spatial correlations of a pixel to its surroundings. To address the aforementioned issues for the task in hand (i.e., segmentation of depolarization image of RF ablated tissue), an “ideal” segmentation algorithm should have the following features:

- The membership of a given pixel to a specific class/segment is spatially correlated to the membership of its neighboring pixels.
- The cut-off values (thresholds) of each class are flexible so that the local membership of a given pixel towards each class will be accounted for in the final segmentation.

The recently proposed segmentation methodology by Aja-Fernandez *et al*. [30] (local fuzzy thresholding algorithm) implements these features. We used this algorithm for depolarization image segmentation. A brief description of the steps involved in this algorithm are given below, while a detailed discussion can be found elsewhere [30].

*Pre-processing*: The input intensity image I(r), where r = (x, y) is position vector indicating pixel location, is smoothed using convolution with Gaussian filter.

*Selection of Number of Segments*: The number of desired segments L (i.e., classes) of the input image I(r) (L = 3 in our case, with l_1_ = RFA core, l_2_ = RFA rim and l_3_ = healthy segment) are defined by the user.

*Fuzzy Membership Functions*: A fuzzy membership function is assigned to each image segment l on the basis of their histogram h(l). Every pixel in the input image is assigned a membership degree [μ_1_(l(r)), μ_2_(l(r)),…,μ_L_(l(r))] with each output segment via the fuzzy membership function. This step is the first major difference from the global rigid thresholding where a pixel’s classification is binary, solely based on its absolute value. Here, the *initial* fate of a pixel to a given segment is decided by its maximal association arg*max* [μ_1_(l(r)), μ_2_(l(r)), …, μ_L_(l(r)), towards that segment. The sum of all *l* weighted membership functions is given as

$$h\;(I)\approx \sum_{l=1}^{L}w_{l}p_{l}(x;{ɵ}_{l})$$ 1

where p_l_(x;ɵ_l_) is the Gaussian probability function with ɵ_l_ parameters (i.e., mean and variance) of the distribution, x is the pixel value (i.e., intensity) and w_l_ are the weights which satisfy Σ w_l_ = 1.

*Neighbourhood Aggregation*: For a given pixel, information about the membership degree of its neighbours is collected. The original membership degree of the pixel is then modified using the local/surrounding membership degrees of its neighbouring pixels. This step is the second distinctive feature of the local fuzzy thresholding methodology.

*Image Segmentation*: The modified membership functions are used to calculate the final image segmentation of each pixel as belonging to l_1_, l_2_ or l_3_.

### Ground truth segmentation

Manual segmentation on histological images after the 4-μm-thick slides were stained and scanned was performed by a pathology expert (professor of histopathology) who was blind to the automated polarimetry segmentation results. The expert manually delineated the lesion core and rim boundaries based on appearance, shape, and size of cardiomyocytes in the RFA lesion compared to their normal morphology in healthy tissue. Specifically, changes in microscopic cellular features such as loss of normal elongated structure of cardiomyocytes, cellular desiccation and shrinkage, cytoskeleton damage and increased color intensity (dark-reddish hue) were considered. Closed contours encircling the RFA core and rim were drawn, thus separating the tissue into three distinct segments for comparison with results of the polarimetric local fuzzy thresholding algorithm.

### Performance evaluation of local fuzzy thresholding algorithm

Various criteria have been proposed to assess performance of image segmentation algorithms. Dice similarity coefficient (DSC) is one of the widely used assessment metrics for medical image segmentation [31,32] and was calculated in this study.

DSC quantitatively measures spatial agreement between two image segmentation schemes. Typically, DSC is defined as the ratio of intersection to union of algorithm-based (A) and ground truth (GT) segmentations.

$$DSC=\frac{2(GT\cap A)}{\left(GT\cup A\right)}$$ 2

where ⋂ and ⋃ represent the intersection and union, respectively. DSC values vary from 0 to 1, which corresponds to no overlap and perfect congruence between two image segmentation schemes, respectively. Partial agreement is described by intermediate values (0 < DSC < 1). In addition to DSC, other metrics used here for segmentation validation were *sensitivity (S*_*n*_—the intersection between ground truth and automated segmentation over the extent of GT); *specificity (S*_*p*_—the fraction of the non-target (background) pixels over the non-GT pixels); and accuracy *(Acc*—the fraction of correctly classified pixels over the entire image).

## Results

**Fig 2:**
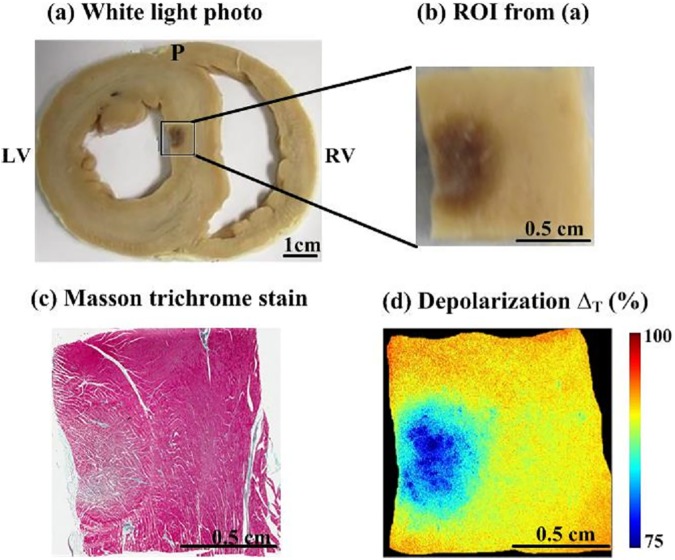
Polarimetry reveals regions of radiofrequency ablation. (a) White light photograph of gross myocardial tissue sample from the septum with RFA lesion, (b) Magnified view of the ROI analyzed with optical polarimetry, the RF ablated region appears dark, (c) Masson’s trichrome histology of the same sample, and (d) Depolarization map of the region analyzed with optical polarimetry. The RFA region had lower depolarization. RV = right ventricle; LV = left ventricle; P = posterior.

Images from a representative porcine myocardial septum sample with RFA lesion (approximately 1 cm^2^ in area) are shown in Fig 2. A magnified view of the region analyzed with optical polarimetry is shown in Fig 2B. The lesion appears dark due to hemorrhage. This is typically seen away from the center of an RFA lesion. Closer to the center, the lesion appears white due to thermal coagulation. (see S1 Fig). Though this leads to significant and variable tissue contrast, it does not clearly delineate the rim region, where there are mixed populations of damaged and viable cardiomyocytes. Masson’s trichrome histology is shown in Fig 2C, where cytoplasm appears pink, nuclei are dark red, and collagen is blue. Histology revealed that healthy myocardium is largely composed of aligned arrays of cardiomyocytes (stained red). Cardiomyocytes within the RFA core region suffered thermal insult and coagulative necrosis; cellular membrane and morphology were structurally altered but maintained some residual architecture. The corresponding depolarization image is shown in Fig 2D. Lower depolarization values were observed in the RFA lesion compared to surrounding healthy tissue. Specifically, the decrease in depolarization was more prominent at the center of RFA lesion, and increased gradually towards healthy tissue at the remote periphery of the lesion. These observed polarimetry changes make sense in the context of the microstructural changes observed with histology, as the thermal coagulation caused by RF ablation results in a homogenization of tissue anisotropy, and hence a loss of linear retardance. A decrease in magnitude and heterogeneous distribution of the anisotropic micro-domains permits light to retain incident polarization to a greater degree, yielding lower depolarization. Depolarization may thus form a useful polarimetric biomaker to delineate RFA lesion extent [22].

**Fig 3:**
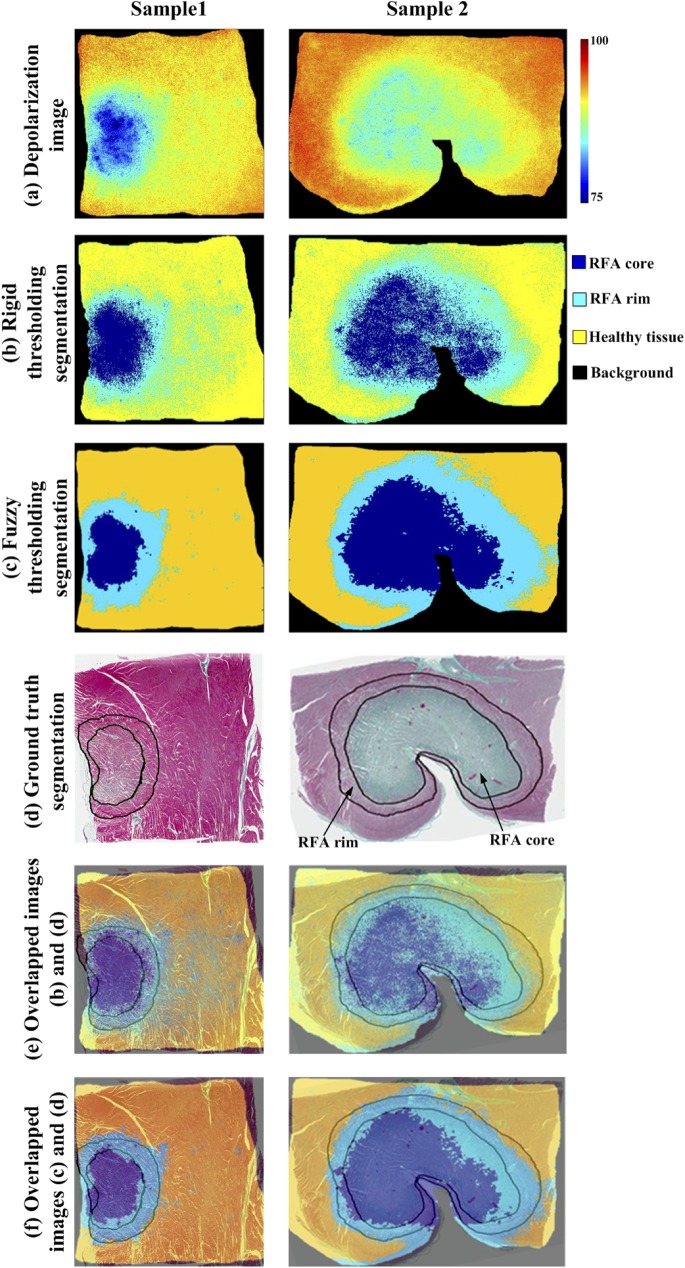
Representative polarization images from two ablated myocardium samples, segmented into RFA core, RFA rim, and healthy regions. (a) depolarization images; (b) automated segmentation with global rigid thresholding; (c) automated segmentation using local fuzzy thresholding algorithm. Pseudo-colors dark blue, light blue and yellow show RFA core, rim and healthy regions, respectively. Black represents the background; (d) segmented histology image (ground truth) where demarcation of RFA core and rim regions are indicated by the black contours; (e) overlapped images from (b) and (d) showing global rigid thresholding and ground truth segmentation; (f) overlapped images from (c) and (d) demonstrating good qualitative agreement of the local fuzzy thresholding and ground truth segmentation scheme. Local fuzzy thresholding shows better qualitative agreement than the global thresholding, which overestimates the extent of the rim region. Quantitative results are summarized in Table 1 and Figs 4 and 5.

**Fig 4:**
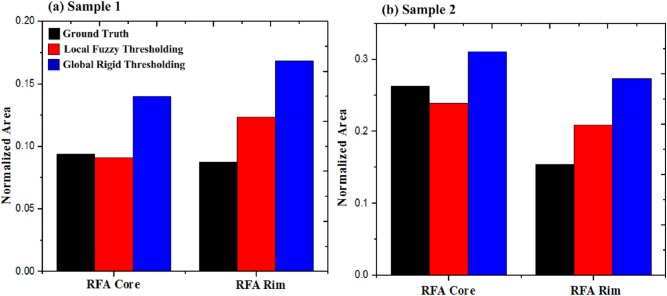
Comparison of normalized area ratios (with respect to total sample area) for RFA core and rim segments as calculated using ground truth (black), local fuzzy (red) and global thresholding (blue) algorithms. Results are shown for (a) Sample 1 and (b) Sample 2. The local fuzzy thresholding algorithm demonstrates better area agreement with the ground truth segmentation than did the global rigid thresholding. It is worth noting that performing identical image pre-processing before global rigid thresholding did not improve the results.

**Fig 5:**
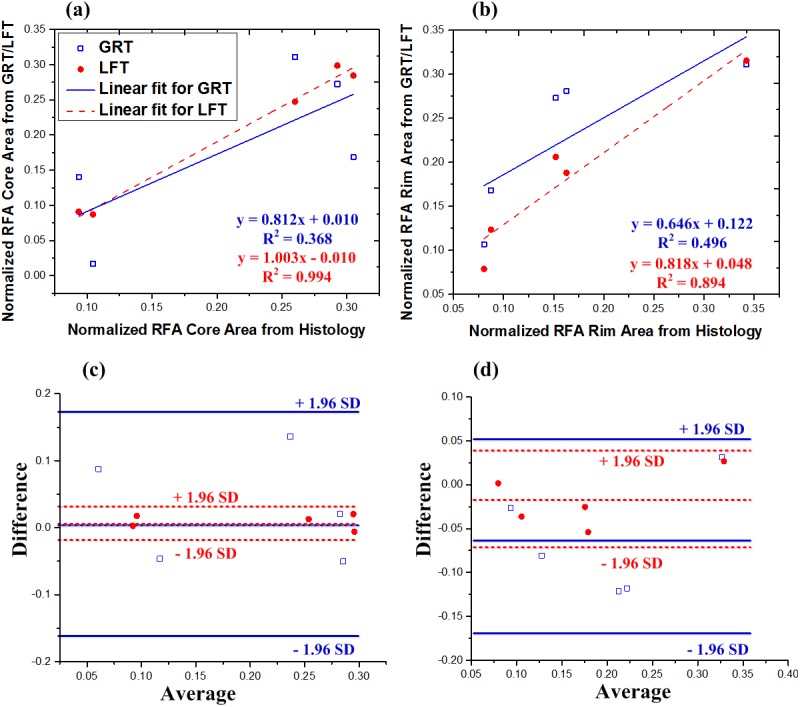
Comparison of both thresholding algorithms with ground truth segmentation of RF ablated tissue. Normalized segment area calculated from global rigid thresholding (GRT, blue hollow squares) and local fuzzy thresholding (LFT, red solid circles) plotted against normalized segment area from ground truth histopathology, for (a) RFA core and (b) RFA rim region. For both RFA core and RFA rim, local fuzzy thresholding provided better correlations. Corresponding Bland–Altman plots for (c) core and (d) rim show no biases (no data points outside +/- 1.96 standard deviations).

Optical polarization images and their corresponding automated and ground truth segmentation results from two representative myocardium samples are summarized in Fig 3. Specifically, total depolarization images from two samples (one from Fig 2) are shown in Fig 3A. Computer assisted segmentation of these optical images into RFA core, RFA rim, and healthy segments with global rigid and fuzzy thresholding algorithms are shown in Fig 3B and 3C, respectively. Mean depolarization values (and corresponding standard deviations) in the healthy, RFA rim and core regions (91.5 ± 1.3%; 87.7 ± 1.4%; 83.3 ± 3.5%, respectively) were used to determine *cut-offs* in the global rigid thresholding [21]. The mean depolarizations were found in 50 × 50 pixel regions of interest (ROIs) for all samples. The location of these ROIs was manually chosen to be near the center of the RFA lesion core, rim, and healthy tissue. The RFA core, RFA rim and healthy regions are represented by the pseudo-colors dark blue, light blue and yellow, respectively (black represents background (no tissue)). The ground truth segmentation was obtained from expert histopathologist demarcation of RFA core and RFA rim regions (indicated by black contours) on the histology image (Fig 3D). Note that global rigid thresholding overestimates the RFA rim region (compared to histopathology) as extending significantly into RFA core and/or healthy regions. Fuzzy thresholding minimizes this overestimation. The fuzzy thresholding segmentation is qualitatively compared with the ground truth segmentation in Fig 3F by overlapping the two images; good agreement was observed. Quantitative metrics for the performance of fuzzy thresholding segmentation for all five samples are summarized in Table 1 and Figs 4 and 5.

**Table 1:** Local fuzzy thresholding algorithm performance. Dice similarity coefficient (*DSC*), sensitivity (*S*_*n*_), specificity (*S*_*p*_) and accuracy (*Acc*) of automated segmentation of total depolarization image compared to ground truth histopathology demarcation of RFA myocardial samples into healthy, rim, and core regions.

RFA Sample	RFA core				Rim region			
	*DSC*	*S*_*n*_	*S*_*p*_	*Acc*	*DSC*	*S*_*n*_	*S*_*p*_	*Acc*
1	0.80	0.89	0.96	0.95	0.77	0.95	0.85	0.88
2	0.76	0.73	0.72	0.73	0.82	0.86	0.62	0.62
3	0.76	0.98	0.83	0.86	0.82	0.96	0.64	0.80
4	0.79	0.72	0.84	0.79	0.81	0.78	0.51	0.68
5	0.77	0.83	0.47	0.70	0.80	0.99	0.99	0.57
Mean ± SD	0.78 ± 0.02	0.83 ± 0.10	0.76 ± 0.17	0.81 ± 0.09	0.80 ± 0.02	0.91 ± 0.08	0.72 ± 0.17	0.71 ± 0.10

To quantitatively compare the expert-outlined (ground truth) segmentation and computer-aided fuzzy thresholding segmentation results of RFA myocardia, we calculated DSC, sensitivity, specificity, and accuracy for both RFA core and RFA rim region segmentations (Table 1). The mean DSC values, representative of the spatial overlap between the ground truth and automated segmentation, for RFA core and rim regions calculated from all five RFA samples were 0.78 ± 0.02 and 0.80 ± 0.02, respectively, which indicate a good agreement between the two segmentation schemes. The mean sensitivity and specificity of the fuzzy thresholding segmentation for the RFA core were 0.83 ± 0.10 and 0.76 ± 0.17, and 0.91 ± 0.08 and 0.72 ± 0.17 for the RFA rim. Further, mean segmentation accuracy values in the RFA core and rim region were 0.81 ± 0.09 and 0.71 ± 0.10, respectively.

Fig 4A and 4B present bar graphs comparing the normalized area of RFA core and rim regions as calculated from ground truth, local fuzzy and global thresholding algorithms for sample 1 and sample 2. Normalized area was defined as the ratio of the given segment (core or rim) area to total area of the sample The local fuzzy thresholding algorithm showed better area agreement with the ground truth segmentation than did the global rigid thresholding.

To quantitatively evaluate the efficacy of both algorithms (for all five samples), Fig 5 shows the correlation of normalized areas calculated with both segmentation algorithms, compared to ground truth histopathology. Results are plotted for each sample, segmented using both local fuzzy thresholding and global rigid thresholding. A perfect segmentation would produce identical normalized areas to that observed with ground truth histology, and thus a slope of unity. Local fuzzy thresholding provided excellent correlation with histopathology for the core segmentation (slope = 1.003) and very good correlation for the rim (slope = 0.818). Despite these good correlations, accurate delineation of the rim region is clearly more difficult compared to the RFA lesion core. Further, local fuzzy thresholding gave superior results to global rigid thresholding, the latter yielding slope of 0.812 for core and 0.646 for rim segmentations. Bland–Altman plots are also shown in Fig 5C and 5D for the core and rim regions, respectively. No biases were observed for either segmentation algorithm (i.e., no data points outside ± 1.96 standard deviations).

## Discussion

The purpose of this study was to quantitatively assess the efficacy of optical polarimetry for delineating RF ablated myocardial tissue into regions of varying thermal damage which are clinically relevant to the treatment of focal cardiac arrhythmia. Differences in Mueller matrix-derived depolarization values were used to segment the polarimetric image into RFA core, RFA rim and healthy tissue regions using a computer-aided methodology (fuzzy segmentation algorithm). The results were compared to, and validated by, ground truth histopathological analysis.

The observed depolarization trends can be interpreted in terms of two major characteristics of myocardial tissue: its scattering and microstructural anisotropy. Coagulation increases the scattering coefficient [33], suggesting increased depolarization. However, tissue structural anisotropy appears to contribute more towards depolarization, as suggested previously [17,34]. Specifically, tissue anisotropy may be spatially heterogeneous; light travelling through micro-domains of spatially varying anisotropy (both in magnitude and orientation) experiences additional randomization of its polarization state, and thus increased depolarization [17]. Indeed, a decrease in linear retardance accompanied by reduced depolarization has been previously observed [35]. For example, anisotropic myocardial tissue exhibits higher depolarization compared to kidney cortex, despite these tissues having similar transport albedos (combination of both scattering and absorption); this indicates that tissue anisotropy contributes more to depolarization than scattering and absorption properties [34]. Likewise, here we postulate that thermal damage of the myocardium results in decreased tissue anisotropy, as reflected in better preservation of light polarization.

Linear retardance information may help segmentation. When used on its own, it was unable to distinguish between the rim and core regions with sufficient statistical confidence [22]; however, we are currently investigating ways to improve tissue contrast by combining several polarization results into novel composite polarimetry metrics.

Unsupervised segmentation is a difficult (it should be both clinically acceptable and computationally efficient) but important (because it is fast and non-subjective) part of medical image analysis, where the imaging field is typically composed of several tissue types and/or their pathological states. Here, we compared two different algorithms–global rigid thresholding and local fuzzy thresholding–for multi-region (RFA core, RFA rim and healthy tissue) image segmentation of the optical depolarization images of RF ablated myocardium (Fig 4).

Global rigid thresholding is based on the idea that an image has a multimodal (tri-modal in our case) histogram that can be exploited to separate different objects of the image using cut-off values. To this end, mean depolarization values of RFA core, RFA rim and healthy tissue were used as global rigid thresholds for automated delineation into these three clinically relevant zones. However, such segmentation failed to properly delineate the RFA rim. Specifically, the global rigid thresholding algorithm overestimated the size of the RFA rim as extending substantially into the RFA core and/or healthy zones, in contrast to the ground truth histopathology. This disagreement was presumably due to the gradual transitions between depolarization levels at the border zones. Further, since global rigid thresholding does not take spatial correlation into consideration, and the absolute depolarization differences between the three regions is small, image noise may lead to *isolated* pixels being incorrectly classified, as indicated in Fig 3B.

The *hard* pixel assignments in global rigid thresholding (i.e., a pixel belongs to or does not belong to a specific class (segment)) is replaced by *soft* assignment in local fuzzy thresholding; the membership degree of a given pixel is modified in relation to its surrounding pixels. This implicitly resulted in a locally variant threshold for image segmentation. The depolarization image segmentation with such adaptive (fuzzy) thresholding delineated the image into RFA core, RFA rim and healthy regions. It is noteworthy that the clinically important RFA rim region was depicted here in such a way that the large number of *isolated* pixels (as seen in global rigid thresholding) was almost completely eliminated. The segmentation results were compared with the ground truth segmentation with the help of evaluation parameters such as DSC, sensitivity, specificity, and accuracy (Table 1).

Accurate segmentation and quantification of RFA lesion extent is essential for assessing the success of RFA treatment of cardiac focal arrhythmia. Reasonably good agreement was observed for the core and rim region as suggested by relatively high mean DSC (0.78 ± 0.02 and 0.80 ± 0.02, respectively). Further, automated segmentation of total depolarization image yielded consistently high mean sensitivity for both RFA core and rim region (0.83 ± 0.10 and 0.91 ± 0.08, respectively). Other evaluation metrics (specificity and accuracy) also indicated good agreement between the automated and ground truth segmentation. Collectively, these statistics illustrate the capability of total depolarization along with the local fuzzy thresholding methodology for segmenting the RF ablated tissue into RFA core, RFA rim and healthy zones.

Previously, other optical techniques such as photoacoustic imaging [8] and OCT [9,10] have been used for segmenting RFA lesions. Although OCT has superior resolution and provides depth-resolved images, past work only qualitatively assessed the accuracy of RF lesion segmentation [22]. Quantitative comparison of photoacoustic image segmentation with RFA lesion delineation of nitro-tetrazolium blue gross pathology (ground truth) showed 69% area agreement. Recently, 3D optoacoustic imaging has been explored to assess RFA lesions in freshly excised porcine myocardial tissue. Specifically, the RFA lesion boundary was marked at locations where the optoacoustic signal exceeded an arbitrary threshold (chosen to be a 30% increase) from the original signal. The study claims “excellent agreement” between histology and optoacoustic images for size and geometry of the lesion but did not provide any quantitative evaluation metrics [36]. Further, acoustic radiation force impulse (ARFI) ultrasound imaging has been used for quantifying the dimensions of myocardial ablation lesions. Manual delineation of the lesion based on visible tissue discoloration, compared with automatic segmentations of the ARFI image resulted in maximum lateral and axial mean overlap of 68.7±5.21% and 66.3±8.4% [37]. In addition to optical imaging, conventional medical imaging such as CT has been used to characterize cardiac RFA lesions. Specifically, a comparison of manual slice-by-slice and semi-automatic segmentation of CT images for RFA visualization in the liver resulted in DSC = 0.77 ± 0.04 [38]. Further, the area of cardiac RFA lesions as seen from MR images (55.4±7.2 mm^2^) correlated well with histological necrosis area (49.7±5.9 mm^2^) [4]. Comparison of manual and geometric model-based semiautomatic segmentation of RFA lesion in rabbit thigh resulted in median error of ≤1.21 mm for the core region and ≤ 1.00 mm for the outer hyper-intense rim region [11]. The comparison of the abovementioned studies to the present study is difficult due to different evaluation metrics (i.e., area agreement, overlap, etc. vs. DSC). Nevertheless, previous studies were limited to binary segmentation of RF ablated tissue (RFA core vs. healthy); these techniques could not delineate the clinically important RFA rim region. In contrast, the current study demonstrates total depolarization images can be used for automatic quantitative segmentation of RF ablated myocardia into all three clinically important regions (core, healthy, *and* rim).

The segmented depolarization image was compared with the ground truth segmentation performed using histological images. However, histology processing (tissue paraffin embedding, sectioning, fixing, staining, etc.) can cause shrinking and slight changes in shape of the tissue samples, as reported in many studies. For instance, mean shrinkage of 11.6% in skin samples [39], 6.2% in oral cavity samples [40], and 4.5% in breast samples [41] due to histology processing has been reported. Quantitative characterization of tissue shrinkage (both size and shape) in myocardial samples during histology processing is relatively unexplored despite its potential importance. Such inevitable tissue shrinkage depends on many factors such as tissue type, fixative agent, fixative length, and tissue desiccation during the fixation and dehydration processes. Thus, the tissue shapes of the depolarization and histology images do not match perfectly. We addressed tissue shrinkage by equalizing the size of both histology and polarimetry images, based on the tissue dimensions in the white light photo, using a custom Matlab program. However, the shapes do not perfectly agree. Further, the automated segmentation was performed on depolarization images obtained from measurements of thick (4mm) tissue samples (to better approximate potential clinical scenario), while the ground truth segmentation was done on a thin histology slice (4 μm, from the same tissue); this discrepancy might have contributed towards the observed alterations in tissue shape resulting in slight misalignment of the two images as seen in the overlapping image (Fig 3E). In addition, the shape of histology slice will presumably change over the depth. This discrepancy between the two images may be considered as a limitation of our study. This issue demands a more robust investigation in any future work. Specifically, quantitative methods characterizing RFA lesion extent based on optical polarimetry images and reconstructed histology slice “stacks” could lead to more accurate results. Alternatively, polarimetry measurements on thin histology slices may eliminate the shape discrepancies and enable improved accuracy in image co-registration.

While polarimetry images are not depth resolved *per se*, they do provide a depth-weighted average of tissue polarimetric properties. Simulation results and experimental validations suggest that the average path length of polarization preserving photons for tissues in the visible spectrum is ∼4–6 mm; a typical sampling depth in the back-scattering directions is half of this, or ~ 2–3 mm in most mammalian tissues [42, 43]. Thus, the measured polarimetry images represent depth-weighted composites over such thicknesses. For potential clinical applications, we will make use of average path lengths estimated from our Monte Carlo engine [43], as well as polarimetric depth heterogeneity models that can detect the presence and degree of tissue axial heterogeneity [16].

Histology was implemented on specific thin slices (4 μm) selected from the tissue samples. The histology images will likely change with depth, affecting the comparison with depth-integrated polarimetry signals. Again, future studies should be done with polarimetry on thin samples, or conversely with histology “stacks” throughout an entire thick sample. We are currently working on a detailed polarimetry study of thick vs thin myocardial tissues (accompanied by histology and Monte Carlo simulations) in preparation for clinical work. Future work will also characterize RF lesions created *in vivo*. We expect that blood flow in capillaries (microcirculation) and edema to affect the formation, extent and morphology of the RF thermal ablation lesion.

Formalin fixation is commonly used in histology, and its effect on optical and polarimetric tissue properties has been previously explored by our group [44]. Fixed tissues exhibited a slightly increased scattering coefficient while absorption remained unchanged. Specifically, fixation of rat myocardial tissues resulted in increased linear retardance (~10%) and depolarization (~25%). These overall changes will not limit the ability of optical polarimetry to detect *contrast* arising from thermal damage, as it is the relative changes from thermally-induced differences that we are after. Nevertheless, future studies should be done on fresh tissue, in preparation for clinical work.

This study demonstrates the feasibility of local fuzzy thresholding for objective auto-segmentation of polarimetrically-imaged RFA myocardium into three clinically relevant zones. However, other possible segmentation techniques may also prove useful, and their performance will be compared to local fuzzy thresholding in future studies. For clinical translation, the development of a clinical intracardiac probe capable of concurrent RF ablation and polarimetric imaging is desirable. Indeed, similar integrated probes with combined RFA-optical coherence reflectometry catheters for real time assessment of RFA lesions have been recently reported [45]. Further, construction of optical polarimetric probes for *in vivo* studies has been considered [46]. If successful, a combined RFA-polarimetry probe may allow real time characterization of RFA lesions, possibly reducing the unsuccessful treatment rate (~10%) of ventricular arrhythmias [47–49].

## Conclusion

This study demonstrates the potential utility of optical polarimetry and automatic segmentation for assessing cardiac RF ablation. Specifically, changes in total depolarization were successfully exploited to segment a polarimetry image into RFA core, rim and healthy regions using local fuzzy thresholding algorithm. The automated segmentation results were validated with ground truth segmentation obtained from expert manual demarcation of the histopathology image. Comparison metrics indicated good agreement between the automated and ground truth segmentations. The findings suggest potential of polarimetry imaging, combined with automatic objective segmentation, for visualizing the spatial extent of RFA core, rim, and healthy myocardial tissue.

## Supporting information

S1 Fig Polarization images from remaining ablated myocardium samples, segmented into RFA core, RFA rim, and healthy regions. (a) White light photograph of gross myocardial tissue sample with RFA lesion, (b) Magnified view of the ROI analyzed with optical polarimetry, (c) depolarization images, (d) automated segmentation with global rigid thresholding, (e) automated segmentation using local fuzzy thresholding algorithm. Pseudo-colors dark blue, light blue and yellow show RFA core, rim and healthy regions, respectively. Black represents the background. (f) segmented histology image (ground truth) where demarcation of RFA core and rim regions are indicated by the black contours, (g) overlapped images from (d) and (f) showing overlap of the global rigid thresholding with ground truth segmentation, and (h) overlapped images from (e) and (f) demonstrating good qualitative agreement of the local fuzzy thresholding and ground truth segmentation scheme. Quantitative results are summarized in Table 1 and Fig 5. (RV = right ventricle; LV = left ventricle; P = posterior).
(TIFF)
