# Supplementary figures and images for: Polarization image segmentation of radiofrequency ablated porcine myocardial tissue
Source: PLoS One. 2017 Apr 5;12(4):e0175173. doi: 10.1371/journal.pone.0175173 (PMC5381909; doi:10.1371/journal.pone.0175173)

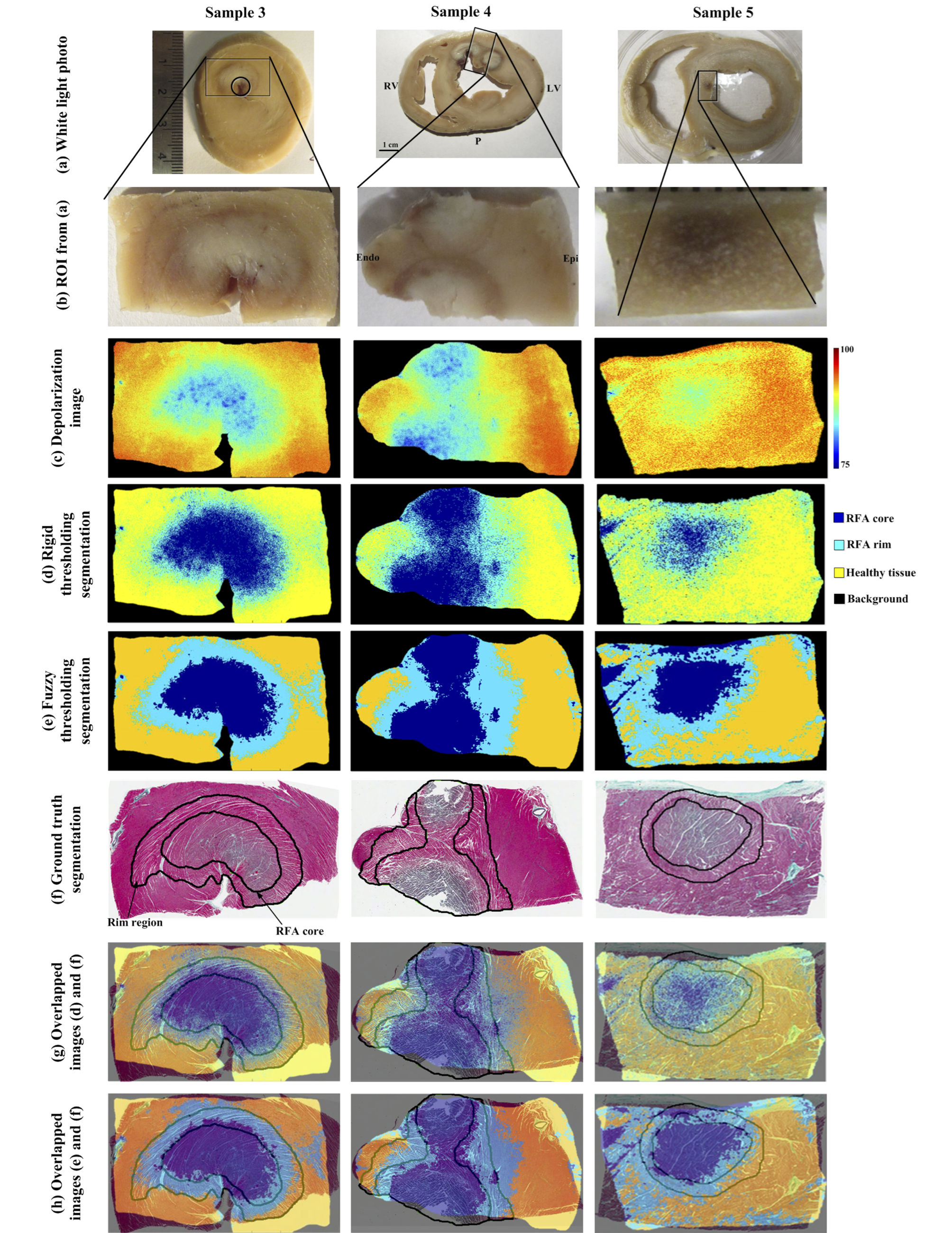

Supplement: S1 Fig — (a) White light photograph of gross myocardial tissue sample with RFA lesion, (b) Magnified view of the ROI analyzed with optical polarimetry, (c) depolarization images, (d) automated segmentation with global rigid thresholding, (e) automated segmentation using local fuzzy thresholding algorithm. Pseudo-colors dark blue, light blue and yellow show RFA core, rim and healthy regions, respectively. Black represents the background. (f) segmented histology image (ground truth) where demarcation of RFA core and rim regions are indicated by the black contours, (g) overlapped images from (d) and (f) showing overlap of the global rigid thresholding with ground truth segmentation, and (h) overlapped images from (e) and (f) demonstrating good qualitative agreement of the local fuzzy thresholding and ground truth segmentation scheme. Quantitative results are summarized in Table 1 and Fig 5. (RV = right ventricle; LV = left ventricle; P = posterior). (TIFF) [file pone.0175173.s001.tiff]
